# Hysteria

**Published:** 1891-11

**Authors:** Fannie Leake

**Affiliations:** Austin


For Daniel's Texas Medical Journal.

### HYSTERIA.

BY FANNIE LEAKE, M. D., AUSTIN.

Read at the Austin District Medical Society, June 20, 1891.

HYSTERIA is a misnomer but received that appellation from a popular idea that it depends on disturbances of the uterus, but of necessity it has no relation whatever to that organ or its appendages. It is a neurosis, and perhaps as nearly as we can define, a condition the intimate pathology of which is as yet involved in so much of doubt and obscurity—is as a functual disturbance of the nervous system characterized by various intellectual, sensory and motor symptoms according to the seat. The extreme variableness of the manifestations and consequent liability to class it with graver disturbances, as well as on the other hand with affectation or the desire to deceive, render the greatest care necessary in diagnosis in doubtful cases. There are often such marked departures from normal functions of various organs as to often engender very perplexing symptoms, and so closely to simulate those common to certain organic pathological conditions as to entertain grave doubts in the premises; if we fail to remember that when the disturbance is only functional, strong emotion may remove the symptoms when perhaps they appear most grave.

Intellect and not emotion should control the race; when emotional activity becomes excessive, then we have the hysterical condition. The higher nervous centers seem principally to be the seat of disturbance, but to some extent also is it extended to the lower or those presiding over automatic phenomena. The inhibiting or controlling power of the higher centres is lost, or, for a time, in abeyance; the convulsive respiratory efforts of a laugh, caused by excitation of the respiratory center, if not inhibited by the higher or intellectual centers, may, as it were, overflow, and extend to other centers in the medulla and as a result general convulsions ensue.

Ætiology—Predisposing causes. Heredity holds the first rank. There is usually some history of neuroses in the preceding generations; oftener it is hysteria than any other, a hysterical mother. Thus is a foundation laid in a congenitally imperfect development of the higher nervous centers. An hysterical parent is *per se* a poor disciplinarian and consequently the training of such would hardly be calculated to develop a well balanced condition of the higher nervous centers.

Hysteria is more frequent in females than in males from obvious reasons. The predisposing causes have been operative in females from the combined action of forces for many ages. With primitive man the paramount business of life being to obtain food and defend himself from wild animals and the more ferocious of his species, bodily strength and vigor was of first importance. The female, owing to her peculiar organization and her functions of maternity, must of necessity be protected in her care and rearing of the species, and as the condition of society necessarily carried with it great respect for physical strength, it carried also a corresponding contempt for physical weakness. The female *mind* might have been just as vigorous, but lack of opportunity, and of the keen whetting of mind on mind, which comes only from vigorous mental contact with our own kind and thus to wear away under timidity, being by the very nature of her surroundings impossible for the female, brought also contempt for her intellectually in the brain of the brother whose own intellect was at that period none too brilliant.

As civilization advanced and men began to have higher aspirations, without looking at the fact that the mother had the most important offices to fill,—without analyzing that "just as the twig is bent is the tree inclined"—held persistently to this time honored relic of a darker age, this contempt for the female intellect, and withheld from her the very thing needed to lay broad and deep the foundation for higher hopes than any yet the human family have dreamed of. A thorough, systematic education, that she, too, might have a well balanced head, a mind capable of sustaining a lengthened train of reasoning, and thus fit her as a good disciplinarian, suitable to lay the foundation in correct home-training for grand and noble future generations. This was reserved for the present generation of magnificent manhood, and among these advanced thinkers, I am proud to say, the medical fraternity leads, as they have ever led in whatever was of good in the race, whether it was to face death in the pestilence, or in scientific research. Charity at the bedside of the sufferer whom poverty claims as her own, or in the gilded or fashionable pew, or to aid right royally the preparing of a higher type of motherhood in a more systematic education of the female position of the human family.

With this lack of respect for the female mind evolution has worked its slow progress until a higher type of manhood was evolved, and only within the last two decades may it be said that her education was more than simply to develop hysterical conditions.

Until the last twenty or twenty-five years, what has constituted her higher (?) education? Music, painting, dancing and the drama—only those things calculated to develop the emotions, never the reasoning faculties.

In early childhood we rarely see manifestations; then the education and condition of the sexes is nearly similar until about puberty, when, with the advent of increased general activity of all the functions of the body, the girl begins her artificial life with pinched feet, compressed viscera and bodily inactivity, learning accomplishments (?) and to beautify her complexion, etc., while the boy is fitting *himself* for life's stern conflicts with little care for personal appearance beyond cleanliness. Now, if we add to this ill directed education, luxury, leaving no stimulus for mental energy, and a life without a purpose, with all these predisposing causes acting from the "time whereof the memory of man runneth not to the contrary" until so recently, can any intelligent mind wonder that we have so many hysterical females?

Exciting or determining causes: With the foundation laid in heredity, or any long continued cause, then anything to distress or irritate the nervous system, will add the exciting cause, as depression from losses of property or friends, unhappy surroundings, family troubles, in fact, any shock to the nervous system either physical or moral; sometimes the exciting cause is trifling indeed; it may be a disturbance in the ovarian or uterine area, but psychical disturbances are just as apt to precipitate a paroxysm.

Symptoms differ according to area disturbed, whether mental, sensory or motor. When mental, they laugh and cry easily and often misplace them, laugh when they should cry and vice versa. Also hallucinations, delusions and illusions; looking into vacancy and seeing something, and again, these illusions become matters of belief or delusions which cannot be dissipated by the other senses.

Some perversions of perception seem very common in hyperæsthesias and anæsthesias; it is probably sensory *perception* and not the *function of the sensory* apparatus, which is in abeyance; thus an individual may use a needle in sewing with fingers which may be pricked with impunity, the act not being felt or perceived. In disturbances of *sensory area* have hyperæsthesias and anæsthesias mostly of the left side, and they are very valuable from a diagnostic point, and sometimes very remarkable; if the patient's attention is diverted, can often stick needles into the flesh and they do not appear to feel. The anæsthesias of the fauces and absence of faucia reflex, and certain points of hyperæsthesia, one just below the left mammary gland, one at epigastrium, and another over the spinal column below the lower part of the scapular area, and one in the left ovarian region, are rarely absent and may be pretty confidently relied on in diagnosis; also a sense often of suffocation, or as they express it "a knot or ball in the throat," the "globus hystericus." There being no trophic disturbances we exclude lesion of the peripheral nerves themselves. There are also various disturbances of the special senses, as partial loss of sight, hearing, taste or smell, but the capricious character of their appearance and recurrence exclude organic pathological processes.

MOTOR: The symptoms of motor area are local spasms; convulsions, more or less general, and paralysis.

Among local spasms those involving the respiratory muscles are perhaps the most interesting class, especially if there is, as sometimes occurs, a hypersecretion of mucus, and, at the same time, such disturbance of the digestive functions or of assimilation as to produce a tendency to emaciation, it will require care and continued observation to differentiate from phthisis. We often hear from one of these charasteristic classes of patients the remark, "I reckon I've had almost every disease; I was given up by the doctors once to die of consumption, and now I have better health than I ever had before." Careful observation will, however, reveal the fact that the cough does not occur when asleep, or quite alone, or no one in hearing, of whom the patient is cognizant. The cough itself is sharp, ringing, and usually without expectoration. It is greatly influenced by moral causes, as at the inquiry and sympathy of friends it will increase. Chronic spasms of certain muscles occasionally simulate the pulsations of an aneurism and tonic spasm of one or more muscles sometimes resists the action of chloroform, unless pushed to extreme anæsthesia; and of this class those involving the abdominal muscles are most likely to give us trouble in diagnosis, as they are sometimes mistaken for growths in the abdominal cavity. Convulsions are very like those of epilepsy, except that in hystero-epilepsy the consciousness is not entirely lost. The spasm is tonic, as a rule very little clonic. The eyes shut with moving of the ball and twitching of the eyelids. Paralysis common, and may be bedridden for years without wasting, and after months respond to electrity, developing slowly, and at times cured suddenly by profound mental impression (as by the metaphysicians). Contractures also very like spinal disease, but respond to electrical reaction, and absence of atrophy excludes organic lesions. In true epilepsy the patient is insensible, pupils fixed, eyes staring. Spasm, first tonic, then clonic, and in spinal disease we do have atrophy of the affected muscles.

Diagnosis is based on the points just enumerated.

Prognosis is favorable to life but apt to run through almost every phase of the disorder, and sometimes ends in mania or melancholia. The tendency, however, is to cessation after the menopaus or climateric period.

TREATMENT: Medicine is of little avail, as every practitioner has realized, for these are numbered among his most unsatisfactory class, and from that number which is continually shifting from one doctor to another, and no confidence in any of them.

But what has been said of the cause will outline the *treatment.* It should be prophylactic and begun in the cradle, if possible, by teaching self-control, but it is hard for a mother lacking this to impart it to another. As the next best place of beginning will be to develop the future mothers by systematic training of intellect in advanced schools, and in technical schools, also in gymnasiums to develop the muscles. Education and correct reasoning will go farther than any one thing to remove it.

During contractions or convulsions then some powerful impression, as dashing cold water over the patient or a hypodermic of apomorphia, which diverts her mind to another channel. They are generally in poor nutrition, constipated, with sluggish action of the skin and tendency to retention of waste products of tissue metamorphosis; then laxatives, diurectics, diaphoretics, salt water bathing, and after, get rid of products of wash, then build up with iron, quinine, strychnia, and electricity. Above all, occupation; give them something else in life besides themselves to think of. Stimulate them to have some definite object in life, and you will have closed the doors to many imaginary ailments, which often come from lack of something better to occupy the mind.

